# Supplementary material for: Implementation of gender-based violence screening guidelines in public HIV treatment programs: A mixed methods evaluation in Uganda
Source: PLOS Glob Public Health. 2024 Jun 4;4(6):e0003185. doi: 10.1371/journal.pgph.0003185 (PMC11149855; doi:10.1371/journal.pgph.0003185)
Supplement: Attachment — Submitted filename: March 20 2024_Point-by-Point Response to Reviewers_PLOS.docx [file pgph.0003185.s002.docx]

Drs. Catherine Kyobutungi and Madhukar Pai

Editors in Chief

PLOS Global Public Health

March 19, 2024

Dr. Kyobutungi and Pai

Ref. No.: PGPH-D-23-00915 entitled “Implementation of gender-based violence screening guidelines in public HIV treatment programs: A mixed methods evaluation in Uganda”.

We thank you for the opportunity to submit a revised iteration of this manuscript. We greatly appreciate the time and effort that the reviewers invested providing feedback and thoughtful suggestions for improvement. My team and I have worked together to carefully review these comments and have revised the manuscript taking them into consideration. Our responses are articulated in a point-by-point fashion below. Edits that were made directly into the manuscript are outlined in the attached track changed document.

It is our belief that the revised manuscript sufficiently addresses the reviewers’ concerns. We hope that you find our PLOS Global Public Health. We thank you and the Referees once more for your careful review and constructive critique, which has improved the quality of our manuscript significantly.

Thank you for your careful consideration,

Reviewers' comments:

**Comments to the Author**

5. Review Comments to the Author

Please use the space provided to explain your answers to the questions above. You may also include additional comments for the author, including concerns about dual publication, research ethics, or publication ethics. (Please upload your review as an attachment if it exceeds 20,000 characters)

Reviewer #1: 1) The sample size, comprising 12 clinics, might restrict the ability to generalize to the district and national levels. It's important to note that there is a total of [mention the total number] ART clinics within the Wakiso and Kampala Districts of Uganda, for context. However, despite this limitation, the study provides valuable insights into the specific clinics and offers an extension of understanding within the district.

*We thank the reviewer for their feedback. We have provided additional context to the Materials and Methods section from lines 134-135 which we believe elaborates upon the concern as requested by the reviewer.*

2)The clarity regarding the utilization of a questionnaire or checklist as screening tools for GBV and the methodology for segregating the prevalence of GBV among HIV clients is lacking. It is essential to include details about the specific checklist employed and the categorization of identified GBV cases. It's particularly helpful if the study refers to any recommended standardized checklist. This would enhance the comprehension of the methodology and data collection process.

*We thank the reviewer for their feedback. We have provided additional context to the Materials and Methods section from lines 191-197 which we believe elaborates upon the concern as requested by the reviewer.*

3) It's not explicitly clarified the types of services, professionals, or trained staff to which clients were referred. Further elaboration is required for comprehensive understanding.

*We thank the reviewer for their feedback. We have provided additional context to the Materials and Methods section from lines 160-163 which we believe elaborates upon the concern as requested by the reviewer.*

4) The study lacks a comparison between pre-COVID-19 and post-COVID-19 screening periods, which could offer valuable insights. Examining this comparison could be particularly intriguing, given reports from various studies indicating an increase in GBV cases during lockdowns, especially in terms of physical and mental violence by intimate partners. Exploring any potential influence of COVID-19 on incidence rates could provide a comprehensive understanding of the situation.

*We thank the reviewer for sharing this perspective. Unfortunately, we lacked robust data to conduct an in-depth quantitative evaluation of data comparing pre- and post-COVID GBV screening and referral estimates. To the extent possible we attempted to quantitatively speak to the difference before/during pandemic in lines 253-255 and we qualitatively provide insights regarding the impact of the COVID-19 pandemic in lines 501-555.*

5) The study outlines factors affecting GBV screening, like awareness, client volume, and engagement. Providers and partners acknowledge benefits like justice and safety from referrals. But, the study doesn't explicitly link or weigh these factors. The impact of financial constraints and socio-cultural norms on referrals lacks clarity. Clarifying these connections would strengthen the study's significance.

*We thank the reviewer for highlighting this opportunity to bring additional clarity to this manuscript. We have provided additional context throughout to specify the connection between financial constraints/socio-cultural norms and referrals to additional services in lines 359-362 and 382-384.*

Reviewer #2: The manuscript and the ways of analyzing the data used were very good, the information from the research ethics committee was available, the data is great for creating public policies in the region to tackle gender violence.
